# Impact of Sampling Strategy and Population Model on Bayesian Estimates of Vancomycin AUC in Patients with BMI > 40 kg/m^2^: A Single-Center Retrospective Study

**DOI:** 10.3390/medicines12040024

**Published:** 2025-09-30

**Authors:** Sarah A. Ekkelboom, Soraya M. Hobart, Laurie J. Barten, Staci L. Hemmer

**Affiliations:** 1 Community Medical Center, Missoula, MT 59804, USA; sarahekkelboom@bitterroothealth.org (S.A.E.); lbarten@communitymed.org (L.J.B.); 2 Department of Pharmacy Practice, Skaggs School of Pharmacy, University of Montana, Missoula, MT 59812, USA; soraya.hobart@umontana.edu

**Keywords:** vancomycin, Bayesian software, area under the curve, pharmacokinetics

## Abstract

**Background/Objectives**: Growing evidence supports the use of a single trough concentration, rather than both a peak and trough, to estimate the 24 h area under the curve (AUC_24_) of vancomycin using Bayesian software (InsightRx^®^ Ver.1.71). However, patients with body mass index (BMI) ≥ 40 kg/m^2^ are underrepresented in validation studies. Studies in patients with obesity have produced mixed results, potentially because of different population models used. **Methods**: This single-center, retrospective study evaluated adult inpatients with BMI ≥ 40 kg/m^2^. Steady-state AUC_24_ estimates generated by Bayesian software using both two-concentration and one-concentration inputs were compared. Agreement was defined as a percent difference within ±20%. Subgroup analyses were conducted for patients with defined peak and trough concentrations and for comparisons between two Bayesian population models (Carreno vs. Hughes). Linear regression assessed covariates associated with percent difference. **Results**: Among 82 encounters, 97.5% of one-concentration estimates based on the smaller concentration were within ±20% of the two-concentration AUC_24,SS_ (mean difference: 2.9%, 95% CI: 0.14 to 3.8%). Similar agreement was observed using the larger concentration (97.5%, mean difference: −3.1%, 95% CI: −4.7 to −0.1.5%). Subgroup analysis for encounters with true peak/trough levels (*n* = 22) also showed 100% agreement within ±20%. The percent difference did not correlate with BMI or other covariates. Comparison of Hughes vs. Carreno models showed larger variability (only 59.1% within ±20%). **Conclusions**: In patients with BMI ≥ 40 kg/m^2^, Bayesian AUC_24,SS_ estimation using a single vancomycin concentration is feasible. Greater caution is warranted in the setting of acute kidney injury, poor model fit, or targeting AUC at the extremes of the therapeutic range. The population model used to generate the Bayesian AUC estimate has a much greater influence than the number of concentrations analyzed. Furthermore, measuring two concentrations does not ensure concordance between models.

## 1. Introduction

Vancomycin remains a mainstay of therapy for the treatment of methicillin-resistant *Staphylococcus aureus* (MRSA) infections. To minimize the risk of nephrotoxicity and to ensure therapeutic effectiveness of vancomycin in the treatment of severe MRSA infections, therapeutic drug monitoring (TDM) is recommended to maintain the 24 h area under the curve (AUC_24_) between 400 and 600 mg·h/L [1]. According to the 2020 consensus guideline for vancomycin monitoring, the preferred method for estimating AUC_24_ is through analysis of peak and trough concentrations using Bayesian software programs [1]. Measuring two concentrations, rather than one, requires additional resources in staffing, incurs additional laboratory costs, and can negatively affect patient satisfaction. The guideline acknowledges that AUC_24_ estimates via Bayesian analysis of a single trough concentration may be sufficient in some populations; however, the adequacy of a single-level is uncertain in patients with obesity [1].

Patients with a body mass index (BMI) greater than 40 kg/m^2^ are not well represented in studies that support single-level, Bayesian estimates of vancomycin AUC_24_ [2,3,4]. Investigations of trough-only AUC estimates specifically in populations with obesity using Bayesian analysis have produced mixed results [5,6,7]. A variety of a priori models and fitting procedures were used in these studies, suggesting that the accuracy of AUC_24_ estimates based on a single concentration may depend on the population model or the fitting routine utilized in the Bayesian analysis. The 2020 consensus guideline does not identify a preferred a priori model for Bayesian estimation in patients with obesity, nor does it recommend a process for model development [1].

Recent comparisons of different a priori models in patients with obesity have focused on the accuracy in predicting individual concentrations, typically random or trough concentrations, rather than AUC_24_ values [8,9]. From these comparisons have emerged two models with better predictive accuracy in patients with BMI ≥ 40 kg/m^2^, the Hughes model and the Carreno model, with the Hughes model supported by a large dataset (47,965 patients with BMI ≥ 40 kg/m^2^) [8,9]. In this large retrospective review by the makers of InsightRx^®^ (San Francisco, CA, USA), the a priori predicted concentrations of the Hughes model were within 20% of the measured concentrations more frequently than the other ten models, reaching statistical significance in 38 out of 40 comparisons across four age groups. To date, no studies have evaluated the agreement between one- and two-concentration AUC_24_ estimates using the Hughes model.

The purpose of this retrospective study was to characterize the level of agreement between two-concentration and one-concentration AUC_24_ estimates in a population with BMI ≥ 40 kg/m^2^, using commercial software and the Hughes model as the population model in the Bayesian analysis. In addition, the study aimed to characterize the impact of the population model (Carreno vs. Hughes) on the two-concentration Bayesian AUC_24_ estimates.

## 2. Materials and Methods

A single-center, retrospective reanalysis of vancomycin concentrations in adults was conducted at a community hospital in Missoula, Montana, to evaluate the feasibility of monitoring vancomycin therapy with a single concentration in patients with BMI ≥ 40 kg/m^2^. In February 2021, the pharmacy-led vancomycin protocol was updated to recommend measurement of two vancomycin concentrations in the first 48 h for patients with BMI ≥ 40 kg/m^2^. Only one concentration was measured for patients with BMI < 40 kg/m^2^. Per protocol, both concentrations were to be analyzed with a Bayesian software program (InsightRx^®^), and a dosing regimen was to be ordered to achieve a 24 h, steady-state AUC (AUC_24,SS_) between 400 and 600 mg·h/L. Throughout the study period, concentrations were fit using the maximum a posteriori (MAP) Bayesian fitting routine with a linear gradient weighting scheme for measured concentrations.

Measurement of true peak and trough concentrations was not required by the protocol, which allowed pharmacists to order one concentration with morning labs, drawn approximately at 0500. Because vancomycin doses at the hospital are ordered at standard dosing times of 0900 and 2100 (except for the first dose), the morning lab draw is often a random level in the middle of the dosing interval. When a random concentration was drawn with morning labs, the protocol did not dictate the timing of the second vancomycin concentration. Frequently the second level would be ordered closer to a peak or a trough concentration. Pharmacists also had the option of ordering a true peak concentration and a true trough concentration, which was more consistent with the 2020 consensus guideline [1]. The concentration obtained at the shortest post-dose interval was designated the “large concentration,” whereas the concentration at the longest post-dose interval was designated the “small concentration.” Serum vancomycin concentrations were determined by particle-enhanced turbidimetric inhibition immunoassay (Atellica CH Vancomycin Assay, Siemens Healthineers, Tarrytown, NY, USA) on the Atellica CH Analyzer.

Patients were eligible for inclusion if they received an order for “vancomycin per pharmacy” after implementation of the new protocol in February 2021 but before end of data collection on 28 February 2025. Patients in the pediatric, obstetric, labor, or neonatal intensive care units were excluded. Other exclusion criteria included BMI < 40 kg/m^2^, fewer than two vancomycin concentrations measured, renal replacement therapy, transfer from an outside facility with uncertain dosing prior to admission, suspected laboratory errors, levels drawn during vancomycin infusion, or greater than 24 h between measured concentrations.

In the patient record in InsightRx^®^ (ver 1.71), all doses, levels, and serum creatinine concentrations recorded after the second vancomycin concentration were removed from the record. Doing so ensured that the AUC_24,SS_ estimate was determined only from information available up to the measurement of the second vancomycin concentration. The AUC_24,SS_ estimated from the analysis of both concentrations, AUC_24,SS_ (two-conc), was recorded using the dosing regimen ordered by the pharmacist at the time the concentrations were first assessed. The larger concentration was then removed from the analysis to determine the AUC_24,SS_ estimate based on only the smaller concentration, AUC_24,SS_ (small-conc). Similarly, AUC_24,SS_ (large-conc) was determined by removing the smaller concentration from the analysis. The quality of the fit reported by InsightRx^®^ (good, intermediate, or poor) was noted for each of the three analyses.

During the period of data collection from February 2021 to February 2025, the Bayesian population model used by InsightRx^®^ to estimate the AUC_24_ transitioned from the Carreno model to the Hughes model. The Hughes model became the hospital standard in July of 2023. Therefore, when recording AUC_24,SS_ values from existing records created before July 2023, it was necessary to change the population model to the Hughes model. When available in the patient medical record, the AUC_24,SS_ (two-conc) using the older Carreno model was also recorded for comparison with the AUC_24,SS_ (two-conc) using the newer Hughes model. The two models could not be compared for all patients because the Carreno model is no longer available in InsightRx^®^, so vancomycin dosing encounters after July 2023 were not included in this subgroup analysis.

The percent differences between the two-concentration and one-concentration estimates of AUC_24,SS_ were calculated using the following equation

$$percent\;difference=\frac{{AUC}_{24,SS}\left(two\;conc\right)-{AUC}_{24,SS}\left(one\;conc\right)}{{AUC}_{24,SS}\left(one\;conc\right)}\times 100\%$$

Similarly to other studies, the agreement between the two-concentration and one-concentration estimates of AUC_24,SS_ was characterized using the proportion of encounters for which the percent difference was between −20% and 20% [8,10], with 95% confidence intervals computed using the Wilson score method. A percent difference of ±20% represents the maximum difference for which the two-concentration and one-concentration AUC_24,SS_ estimate could both be in the therapeutic range of 400–600 mg·h/L (i.e., if the one-concentration AUC_24,SS_ is 500 mg·h/L and the two-concentration AUC_24,SS_ is either 400 or 600 mg·h/L). The large concentration AUC_24,SS_ estimates and the small concentration AUC_24,SS_ estimates were analyzed separately.

As a secondary analysis of bias, paired AUC_24,SS_ values were compared with a two-sided Wilcoxon signed-rank test (α = 0.05), while the ±20% percent-difference criterion remained the primary agreement metric given the 400–600 mg·h/L therapeutic window. Correlation between the two-concentration and one-concentration AUC_24,SS_ estimates was also assessed using linear regression and Pearson’s correlation coefficient to facilitate comparison with other studies [2,4,5,6,7].

The McNemar–Bowker test was used to compare the distribution of fit quality assessments across the three categories (good, intermediate, poor) when analyzing either two concentrations or one concentration (large or small). Linear regression was used to explore the effect of the following variables on the percent difference between the two- and one-concentration AUC estimates: BMI, weight, age, creatinine clearance, time difference between concentrations, time of large concentration relative to the end of the infusion, time of small concentration relative to the previous dose, and time relative to the start of therapy. Variables with *p* < 0.25 in univariable analysis were entered into the multivariable regression, where significance was defined as *p* < 0.05 for association with percent difference.

Agreement between the two-concentration and one-concentration estimates of AUC_24,SS_ was also investigated for the subgroup of encounters for which both peak and trough concentrations were measured. A peak concentration was defined as a level measured at a time after the end of the infusion that was less than 25% of the dosing interval. A trough concentration was defined as a level measured at a time after the dose that was greater than 75% of the dosing interval. Standard peak (1–2 h post-infusion) and trough (1 h pre-dose) definitions were intentionally broadened to maximize inclusion of dosing encounters with substantial differences in concentration values. In addition, the percent difference between the two-concentration AUC_24,SS_ estimate using the Hughes model and the two-concentration AUC_24,SS_ estimate using the Carreno model was calculated and agreement was characterized using the proportion of differences within ±20%.

Descriptive statistics and linear regression were calculated using Microsoft Excel 2021. Statistical analyses were performed using R (version 4.5.1; R Foundation for Statistical Computing, Vienna, Austria). To promote transparency in study design, data collection, and analysis, the reporting followed guidelines outlined in the Strengthening the Reporting of Observational Studies in Epidemiology (STROBE) checklist.

## 3. Results

**Figure 1:**
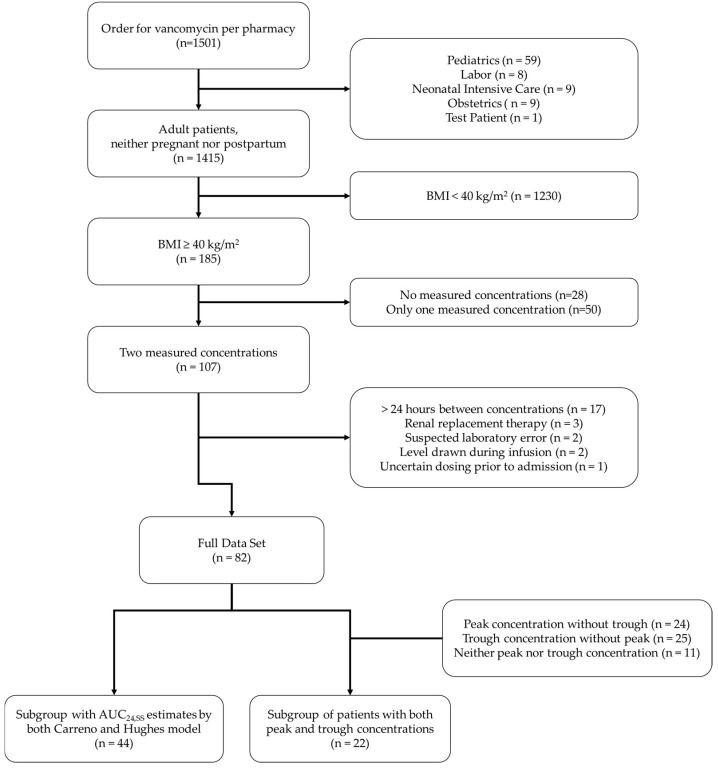
Patient selection and exclusion process for the retrospective reanalysis of vancomycin AUC_24,SS_.

In the four-year period of data collection, the pharmacist-managed vancomycin protocol was ordered during 1501 hospital encounters. Ultimately, 82 different hospital encounters (79 unique patients) were included in the final analysis. Reasons for exclusion are shown in Figure 1, with a BMI < 40 kg/m^2^ being the most common reason for exclusion. Out of the 82 encounters included, 22 encounters (22 unique patients) had both a peak and trough concentration measured. Similarly, 44 dosing encounters (42 unique patients) had an available AUC_24,SS_ (two conc) estimate based on both the Hughes model and the Carreno model.

**Figure 2:**
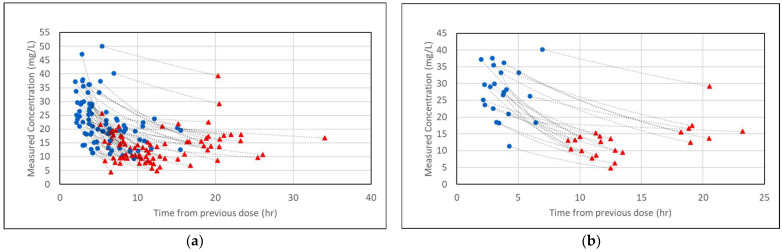
Magnitude and timing of the measured vancomycin concentrations for (**a**) the full dataset and (**b**) the peak/trough subgroup. Two concentrations were measured for each encounter, one larger (blue circles) and one smaller (red triangles). Of note, the “previous dose” may not be the same for the larger and smaller concentrations. Sometimes the smaller concentration was measured first, followed by a dose, then the larger concentration was measured. Exponential trend lines are added to connect concentration pairs and do not represent the actual concentration vs. time curves from the Bayesian fit.

The mean age (SD) of patients in the full dataset was 56.6 (13.5) years with near equal representation of men and women (Table 1). The majority of patients (64.6%) had a BMI between 40 and 50 kg/m^2^. Twenty-two patients (26.8%) had a serum creatinine greater than 1.2 mg/dL at the time levels were measured, with 9 (11%) in acute kidney injury (AKI), defined as a serum creatinine of 0.5 mg/dL or more above baseline. Only 15 patients (18.3%) were critically ill and 7 (8.5%) were diagnosed with septic shock. The mean (SD) daily dose was 2413 (356) mg and the most common dosing interval was every 12 h (63.4%). Using the Hughes model, the median (IQR) AUC_24,SS_ was 522 (477–553) mg·h/L for the two-concentration fit, 511 (473–551) mg·h/L for the small-concentration fit, and 545 (475–592) mg·h/L for the large-concentration fit. The magnitude of the vancomycin concentrations and the time of measurement relative to the previous dose are shown in Figure 2.

### 3.1. Two-Concentration AUC_24,SS_ Compared to the Small-Concentration AUC_24,SS_ Using the Hughes Model

**Figure 3:**
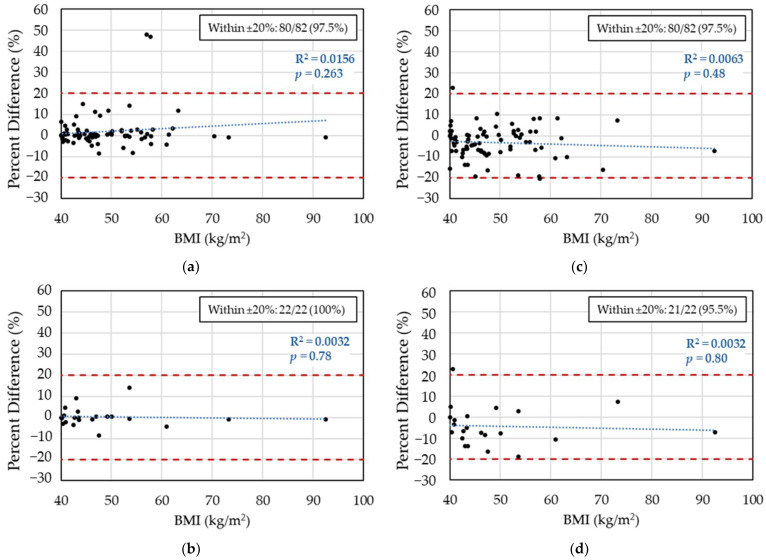
Percent difference between two-concentration and one-concentration AUC_24,SS_ estimates using the Hughes model: (**a**) small concentration, full dataset (N = 82); (**b**) small concentration, peak/trough subgroup (N = 22); (**c**) large concentration, full dataset (N = 82); (**d**) large concentration, peak/trough subgroup (N = 22). Dashed red lines indicate the ±20% agreement range; R^2^ reflects the Pearson’s coefficient from the linear fit (dashed blue line).

The AUC_24,SS_ estimates from the small-concentration fit were strongly correlated with the AUC_24,SS_ estimates from the two-concentration fit with a Pearson’s coefficient of 0.83 (Figure S1a). The mean percent difference between AUC_24,SS_ (two conc) and AUC_24,SS_ (small conc) showed a small but significant bias of 2.9% (95% CI: 0.14 to 3.8%); however, the median AUC values were not significantly different by the Wilcoxon signed rank test (*p* = 0.14). For 97.5% of encounters (95% CI: 91.5 to 99.3%), the percent difference was within ±20% (Figure 3a). Fit quality assessments between the two-concentration and small-concentration analyses were significantly different (*p* = 0.0005, Figure S2). The small-concentration fits were judged be “good” more frequently than the two-concentration fits (97.6% vs. 76.8%) and less commonly graded as “poor” (1.2% vs. 4.9%). Linear regression analysis revealed no apparent relationship between percent difference and patient BMI, *p* = 0.26 (Figure 3a). No other variables were significantly correlated with percent difference in the univariate analysis (Figures S4 and S5). While several variables met the inclusion threshold for the multivariate model (*p* < 0.25), none became significantly associated with percent difference once included (Table S1).

Two encounters had much larger percent differences than the rest of the dataset, over 40%. Specific details about these two patients are shown in Table 2. For encounter #68, the smaller concentration was unexpectedly low at 4.4 mg/L after three doses totaling 3000 mg. Consequently, the fit to the small concentration was judged to be poor by the Bayesian software. For encounter #40, the large concentration was unexpectedly high, producing a poor fit to the two concentrations. However, the fit to the smaller concentration was considered good. On the day concentrations were measured, the serum creatinine was double the baseline value of 0.85 mg/dL, which may have contributed to the intermediate fit quality. Interestingly, the same patient received vancomycin therapy four months earlier and the percent difference between AUC_24,SS_ (two conc) and AUC_24,SS_ (small conc) for the previous encounter was 2.75%. The patient had no evidence of AKI at the previous encounter.

Eight other patients were also experiencing AKI at the time concentrations were measured. For all 8 patients, the percent difference between AUC_24,SS_ (two conc) and AUC_24,SS_ (small conc) was within ±20%. The fit quality assessments from the small-concentration fit were concordant with the two-concentration for 7 out of 8 encounters.

For the subset of encounters with both a peak and trough concentration measured (N = 22), the mean percent difference between AUC_24,SS_ (two conc) and AUC_24,SS_ (small conc) was −0.28% (95% CI: −1.7 to 2.3%). The median difference in AUC values was not significant (*p* = 0.45). The AUC_24,SS_ (two conc) and AUC_24,SS_ (small conc) estimates were more strongly correlated (R^2^ = 0.96) in the peak/trough subgroup than in the full dataset (Figure S1b). For 100% of encounters (95% CI: 85.1 to 100%), the percent difference was within ±20% (Figure 3b). The largest absolute percent difference observed was 14.1%.

### 3.2. Two-Concentration AUC_24,SS_ Compared to the Large-Concentration AUC_24,SS_ Using the Hughes Model

The AUC_24,SS_ estimates from the large-concentration fit were strongly correlated with the AUC_24,SS_ estimates from the two-concentration fit with a Pearson’s coefficient of 0.83 (Figure S1c)_._ The mean percent difference between AUC_24,SS_ (two conc) and AUC_24,SS_ (large conc) was −3.1% (95% CI: −4.7 to −1.5%) and the difference in median AUC values was significant (*p* = 0.0006). However, 97.5% (95% CI: 91.5 to 99.3%) of encounters were within ±20% (Figure 3c). Fit quality assessments between the two-concentration and large-concentration analyses were significantly different (*p* = 0.001, Figure S3). As with the small-concentration fits, the large-concentration fits were judged be “good” more frequently than the two-concentration fits (95.1% vs. 76.8%) and less commonly graded as “poor” (0% vs. 4.9%).

For 8 of 9 encounters with AKI, the percent difference between AUC_24,SS_ (two conc) and AUC_24,SS_ (small conc) was within ±20%. For the other encounter, a percent difference of 22.7% was observed in a patient with septic shock who had a SCr of 1.2 mg/dL at the time of the first measured concentration, which then increased to 2.38 mg/L ten hours after the time of the second concentration. The fit quality assessments from the large-concentration fit were concordant with the two-concentration fit for 7 out of 9 encounters.

Once again, linear regression analysis revealed no apparent relationship between percent difference and patient BMI with a *p*-value of 0.48 (Figure 3c). Three variables were significantly associated with percent difference in the univariate analysis (CrCL FFM, time of trough after dose, time of peak after dose), but when combined with age in the multivariate analysis, no single variable reached statistical significance (Figures S4 and S5, Table S2). However, the overall multivariate model was statistically significant (*p* = 0.002), indicating that the combination of variables explained a meaningful portion of the variance.

For the subset of encounters with both a peak and trough concentration measured (N = 22), the mean percent difference between AUC_24,SS_ (two conc) and AUC_24,SS_ (large conc) was −4.4% (95% CI: −8.5 to −0.3%). The difference in the median AUC estimates was significant (*p* =0.012). The correlation between AUC_24,SS_ estimates did not improve in the peak/trough subgroup (R^2^ = 0.80, Figure S1d). The percent difference was within the range of ±20% for 95.5% of encounters (95%CI: 78.2 to 99.3%), as shown in Figure 3d.

### 3.3. Two-Concentration AUC_24,SS_ from the Hughes Model Compared to Two-Concentration AUC_24,SS_ from the Carreno Model

**Figure 4:**
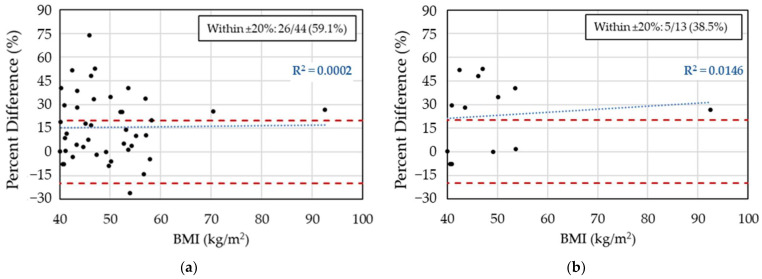
Percent difference in two-concentration AUC_24,SS_ estimates between the Hughes and Carreno models: (**a**) full dataset (N = 44); (**b**) peak/trough subgroup (N = 13). Each point represents the percent difference in AUC_24,SS_ derived from the two models for the same dosing encounter. Dashed red lines indicate the ±20% agreement range; R^2^ reflects the Pearson correlation coefficient from the linear fit (dashed blue line).

The two-concentration AUC_24,SS_ estimates using the Hughes model were only weakly correlated with the two-concentration estimates using the Carreno model (R^2^ = 0.108, Figure S1e). The AUC_24,SS_ from the two-concentration fit using the Carreno model was available for 44 patients. The mean percent difference between AUC_24,SS_ (two conc) using the Hughes model and AUC_24,SS_ (two conc) using the Carreno model was 15.6% (CI: 9.3 to 21.9%). Only for 59.1% of encounters (95% CI: 44.4 to 72.3%) did the percent difference fall within ±20% (Figure 4a). Most out-of-range encounters (17/18) had a positive percent difference above 20%, with a maximum percent difference of 73.7%.

Thirteen encounters in the peak/trough subgroup also had an AUC_24,SS_ (two conc) estimate using the Carreno model. For these 13 patients, the mean percent difference between AUC_24,SS_ (two conc) using the Hughes model and AUC_24,SS_ (two conc) using the Carreno model was 22.9% (95% CI: 9.2 to 36.8%). The percent difference was within the range of ±20% for 38.5% of encounters (95%CI: 17.8 to 64.4%), as shown in Figure 4b.

## 4. Discussion

The purpose of this retrospective review was to determine if a single vancomycin concentration, analyzed with a Bayesian pharmacokinetic software program, would be sufficient to estimate AUC_24,SS_ in patients with a BMI ≥ 40 kg/m^2^. Although measuring both peak and trough concentrations in patient populations with varying volumes of distribution is recommended by the 2020 consensus guideline, this is not always practical. Given the time and resources required to collect vancomycin concentrations in a hospital with limited phlebotomy staff, this study aimed to determine whether the additional lab draw could be eliminated and the timing of a one-concentration monitoring strategy optimized.

The results of this review provide support for estimating vancomycin AUC_24,SS_ with a single concentration. For over 95% of encounters, the difference between the two-concentration and one-concentration AUC_24,SS_ estimates was within 20%. Similar results were observed in the subset of 22 patients for which measured concentrations met peak and trough criteria. On average, the one-concentration AUC_24,SS_ estimate was better when the smaller concentration was analyzed, suggesting that concentrations measured later in the dosing interval will produce more reliable AUC estimates. This might simply be explained by the fact that smaller concentrations have less proportional error and are assigned greater weight in the Bayesian fit [11].

It is important to acknowledge what could be missed with single-level monitoring of vancomycin. For two outliers, the two-concentration AUC_24,SS_ was substantially greater than the one-concentration estimate, which would result in unnecessary vancomycin exposure. One of these patients had rapidly changing renal function, while a surprisingly low concentration was measured in the other patient, resulting in a poor fit. Both the labile renal function and poor fit would have prompted closer examination and more frequent monitoring in these two patients, and it is unlikely that dosing decisions for these patients would have been based on a single level. Measuring a second concentration would be prudent in these situations. A second level is also advisable when dosing at the extremes of the therapeutic range, where the margin for error is narrow.

Single-level monitoring also appears less effective in detecting situations where measured concentrations are not well fit by the Hughes population model. This is not surprising, since two concentrations place stricter constraints on the concentration–time curve and reduce the model’s ability to fit the observed data. The fit quality was judged to be intermediate or poor in 19 (23%) of the two-concentration fits in the full dataset. In the most favorable scenario—when the larger concentration was used—the one-concentration analysis identified an intermediate or poor fit in only 5% of dosing encounters. According to InsightRx^®^, intermediate or poor fits indicate that the measured concentrations are unlikely or very unlikely in the Bayesian population model and should prompt greater scrutiny and/or more intense monitoring. In our sample, 15 encounters (17%) with suboptimal fit would not have been flagged for enhanced review if only a single concentration had been used.

The change from the Carreno model to the Hughes model in the middle of the review period allowed for a comparison of the two-concentration AUC_24,SS_ estimates produced by the two models. The difference in AUC estimates varied widely, with only 59.1% agreement between the two models. On average, the two-concentration AUC estimates from the Hughes model were significantly larger than the two-concentration AUC estimates from Carreno model. Consequently, over 40% of the dosing regimens selected to produce a therapeutic AUC_24,SS_ with the Carreno model would have been considered supratherapeutic by the Hughes model. This finding suggests that the population model used by the Bayesian software is more influential than the number of concentrations analyzed when estimating AUC. Furthermore, obtaining two concentrations does not ensure concordance of AUC estimates across different models developed in patients in obesity. This underscores the need for caution when comparing AUC estimates generated by pharmacokinetic programs that apply different population models.

Our results are similar to those reported by Covington et al. [7]. In their study of 31 patients with BMI ≥ 30 kg/m^2^, the trough-only AUC_24_ estimate was not significantly different from the two-concentration AUC_24_ estimate. For only one patient did the percent difference in AUC_24_ appear to exceed 20% with an absolute difference of 216 mg·h/L. Key methodological differences include Covington et al.’s use of true peak and trough measurements, the use of a one-compartment model in DoseMeRx^®^, and the estimation of AUC_24_ at end of the sampling interval rather than projecting to steady state [7].

Other studies comparing one- and two-concentration vancomycin AUC estimates in patients with obesity have reported significant bias and imprecision with the one-concentration AUC estimate [5,6]. In the first study, a one-concentration model was used to fit the single trough concentration while a two-compartment model was used to fit both concentrations, which could explain the significant difference in AUC estimates [5]. In the second study, three out of the four models tested were not developed in a population of patients with obesity [6]. However, the fourth model (Carreno model) was developed from and tested in 12 patients with BMI ≥ 40 kg/m^2^ using a richly sampled dosing interval (5 levels per patient). For the Carreno model, the mean percent error of the trough-only AUC estimate compared to the reference AUC was substantial (12.5%), with large uncertainty (95% CI: −18.9 to 43.8%). An even larger difference was observed between the mean one-concentration and two-concentration AUC estimates [6].

It is unclear why, in the aforementioned study, the Carreno model produced one-concentration AUC estimates that showed less agreement with the two-concentration estimates. Unlike the model used in our study (Hughes) and the model used in the study by Covington et al. (Adane), the Carreno model was developed using a non-parametric adaptive grid, and the assay variance was modified with an adaptive gamma scalar [7,11,12]. Doing so may have weighted measured concentrations more than the population covariates, resulting in a greater impact on the estimated AUC with additional fit concentrations. Another possible explanation is that the Adane and Hughes models better describe the concentration versus time curve in patients with obesity, because they both include weight as a covariate in the volume of distribution [11,12]. However, recent evidence suggests that the Carreno model is better than the Adane model in predicting vancomycin concentrations in patients with obesity, while the Hughes model outperformed both [8,9].

Strengths of this study include a relatively large sample of patients with BMI ≥ 40 kg/m^2^, exceeding that of similar investigations. This study also reports the agreement between the two-concentration AUC estimate and the peak-only AUC estimate. Previous studies in patients with obesity only reported agreement with the trough-only AUC estimate. Additionally, the comparison was based on projected steady-state AUC_24_ values, which are generally used to guide dosing decisions, rather than pre-steady state values. Sufficient data was also collected to explore the influence of multiple variables on percent difference. Notably, this is the first study of its kind to assess fit quality in both one- and two-concentration analyses.

This study has several limitations. Power calculations were not conducted to determine if the study size was sufficient to reveal a difference between the one- and two-concentration estimates. The definitions of peak and trough concentrations were broader than historical criteria. Even then, only 22 encounters included both a peak and a trough concentration, while 11 encounters had neither. Consequently, the difference between one-concentration and two-concentration AUC_24,SS_ is likely underestimated. In addition, of the 185 eligible patients with BMI ≥ 40 kg/m^2^, 78 were excluded because fewer than two concentrations were measured, which could be a source of bias.

Due to the retrospective design, the study could not assess how one- versus two-concentration analyses would influence actual dosing decisions by clinical pharmacists. Similarly, this retrospective reanalysis of vancomycin levels was unable to assess the impact of single-level monitoring of vancomycin on clinical or nephrotoxic outcomes in patients with BMI ≥ 40 kg/m^2^. Also, software updates over the four-year period cannot be ruled out as a contributor to the difference between the Carreno and Hughes AUC_24,SS_ estimates. However, all user-defined settings, other than the model, remained the same throughout the study period. Finally, this was a single-center study using only one commercial software program and may not be generalizable to other institutions or other software programs.

## 5. Conclusions

In patients with BMI ≥ 40 kg/m^2^, a single vancomycin concentration was sufficient to estimate AUC_24_ in most cases. However, single-level monitoring was less effective at identifying intermediate or poor model fits. The choice of population model had a greater influence on AUC estimates than the number of concentrations used, emphasizing the importance of model selection. Further prospective studies, using different pharmacokinetic software and population models, are needed to confirm these findings and guide dosing strategies in this population.

## Figures and Tables

**Table 1:** Demographic information for patients included in the full dataset (N = 82).

Characteristic	Full Dataset N = 82	Peak/Trough Subgroup N = 22	Carreno Subgroup N = 44
Mean Age (SD), years	56.6 (13.5)	53.3 (13.7)	54.4 (13.3)
Number Male (%)	39 (47%)	8 (36%)	22 (50%)
Number Female (%)	43 (53%)	14 (64%)	22 (50%)
Mean Weight (SD), kg	144.5 (27.4)	141.6 (32)	145.2 (27.6)
Median BMI (IQR), kg/m^2^	46.2 (42.5–53.6)	57.5 (45–64)	57 (45.8–67.3)
BMI categories (kg/m^2^), N (%)			
BMI > 40 and <50	53 (64.6%)	16 (72.7%)	26 (59.1%)
BMI ≥ 50 and <60	22 (26.8%)	3 (13.6%)	16 (36.4%)
BMI ≥ 60 and <70	4 (4.9%)	1 (4.5%)	0 (0%)
BMI ≥ 70	3 (3.7%)	2 (9.1%)	2 (4.5%)
Median serum creatinine (IQR)	0.9 (0.7–1.2)	0.9 (0.7–1.2)	0.9 (0.8–1.2)
Mean CrCL using FFM (SD), mL/min	83 (41.2)	78.9 (31.9)	84.2 (40.7)
Mean CrCL using Adj BW (SD), mL/min	113.3 (54.4)	110.5 (44.2)	114.6 (53.6)
Acute kidney injury within 24 h of level evaluation	9 (11%)	2 (9.1%)	5 (11.6%)
Location, N (%)			
Medical/Surgical Unit	52 (63.4%)	12 (54.5%)	24 (54.5%)
Intensive Care Unit	15 (18.3%)	6 (27.3%)	11 (25%)
Orthopedic Unit	9 (11%)	2 (9.1%)	3 (6.8%)
Progressive Care Unit	4 (4.9%)	2 (9.1%)	2 (4.5%)
Inpatient Rehabilitation Unit	2 (2.4%)	0 (0%)	4 (9.1%)
Dosing interval, N (%)			
Every 8 h	2 (2.4%)	0 (0%)	2 (4.5%)
Every 12 h	56 (68.3%)	16 (72.7%)	28 (63.6%)
Every 24 h	21 (25.6%)	6 (27.3%)	12 (27.3%)
Every 36 h	2 (2.4%)	0 (0%)	2 (4.5%)
Every 48 h	1 (1.2%)	0 (0%)	0 (0%)
Mean of small concentration (SD), mg/L	13.6 (5.6)	12.9 (4.9)	12.6 (4.6)
Median time of large concentration (IQR), as fraction of interval	0.3 (0.2–0.5)	0.3 (0.2–0.3)	0.3 (0.2–0.4)
Median time of small concentration (IQR), as fraction of interval	0.8 (0.6–0.9)	0.9 (0.8–1)	0.8 (0.7–1)
Daily dose after level evaluation (SD), mg	2413 (356)	2341 (1059)	2456 (1386)
AUC_24,SS_ (two conc), Hughes model (IQR), mg·h/L	522 (477–553)	513 (478–603)	530 (483–566)
AUC_24,SS_ (small conc), Hughes model (IQR), mg·h/L	511 (473–551)	505 (485–601)	N/A
AUC_24,SS_ (large conc), Hughes model (IQR), mg·h/L	545 (475–592)	556 (505–649))	N/A
AUC_24,SS_ (two conc), Carreno model (IQR), mg·h/L	N/A	N/A	481 (450–507)

BMI = Body Mass Index; Adj BW = Adjusted Body Weight; FFM = Fat Free Mass; CrCL = creatinine clearance using Cockroft-Gault equation. Adj BW = Ideal Body Weight + 0.4 (Total Body Weight − Ideal Body Weight); If female, FFM = (9270·Total Body Weight)/(8770+244·BMI); If male, FFM = (9270·Total Body Weight)/(6860+216·BMI).

**Table 2:** Information about two dosing encounters that produced percent differences between AUC_24,SS_ (two conc) and AUC_24,SS_ (small conc) of greater than 40%.

ID	Unit	Age (yr)	Sex	Weight (kg)	BMI (kg/m^2^)	SCr (mg/dL)	AKI	Large Concen-tration (mg/L)	Small Concen-tration (mg/L)	Percent Difference Small Conc Fit	Percent Difference Large Conc Fit	Small-Level Fit Quality	Large-Level Fit Quality
40	PCU	41	M	191	57	1.73	yes †	47.1	17.8	47.9%	−6.6%	good	intermediate
68	Med/ Surg	77	F	133.6	57.8	0.99	no	25.6	4.4	46.9%	−19.2%	poor	good

† Serum creatinine (SCr) trend (in mg/dL): Day 1, 1.3; Day 2, 1.89; Day 3, 1.73, Day 4, 1.26, Day 5, 0.89. Concentrations were measured on Day 3.

## Data Availability

The data presented in this study are available on request from the corresponding author due to protect the privacy of patients included in the study.

## Supplementary Materials

The following supporting information can be downloaded at: https://www.mdpi.com/article/10.3390/medicines12040024/s1, Figure S1: Comparison of AUC_24,SS_ estimates obtained using different methods; Figure S2: Distribution of the two-concentration and small-concentration fit quality assessments by the Bayesian software program across the categories of Good, Intermediate, and Poor; Figure S3: Distribution of the two-concentration and large-concentration fit quality assessments by the Bayesian software program across the categories of Good, Intermediate, and Poor; Figure S4: Percent difference between two-concentration and small-concentration AUC_24,SS_ estimates (a–d) and between two-concentration and large-concentration AUC_24,SS_ estimates (e–h) plotted as a function of the following variables: time from therapy initiation, age, weight, and creatinine clearance (N = 82 for all panels); Figure S5: Percent difference between two-concentration and small-concentration AUC_24,SS_ estimates (a–c) and between two-concentration and large-concentration AUC_24,SS_ estimates (d–f) plotted as a function of the following variables: difference in sampling times as fraction of interval, time of small concentration after dose, and time of large concentration after dose (N = 82 for all panels); Table S1: Multivariable linear regression examining predictors of percent difference between two-concentration and small-concentration AUC_24,SS_ estimates. The dependent variable was percent difference, with time of first measurement after initiation, time of trough after dose, and time of peak after dose included as independent variables; Table S2: Multivariable linear regression examining predictors of percent difference between two-concentration and large-concentration AUC_24,SS_ estimates. The dependent variable was percent difference, with age, creatinine clearance (CrCL, fat-free mass adjusted), time of trough after dose, and time of peak after dose included as independent variables.

## Abbreviations

The following abbreviations are used in this manuscript:

Adj BW	Adjusted Body Weight
AKI	Acute Kidney Injury
AUC	Area-under-the-curve
AUC_24,SS_	24-h AUC at steady-state
BMI	Body mass index
CrCL	Creatinine clearance calculated using Cockcroft-Gault equation
FFM	Fat Free Mass
MAP	Maximum a posteriori
MRSA	Methicillin-resistant *Staphylococcus aureus*
SCr	Serum creatinine
