# Supplementary material for: Impact of Sampling Strategy and Population Model on Bayesian Estimates of Vancomycin AUC in Patients with BMI > 40 kg/m2: A Single-Center Retrospective Study
Source: Medicines (Basel). 2025 Sep 30;12(4):24. doi: 10.3390/medicines12040024 (PMC12551005; doi:10.3390/medicines12040024)
Supplement: Supplementary file 1 [file medicines-12-00024-s001.zip › medicines-3834516-supplementary.pdf]

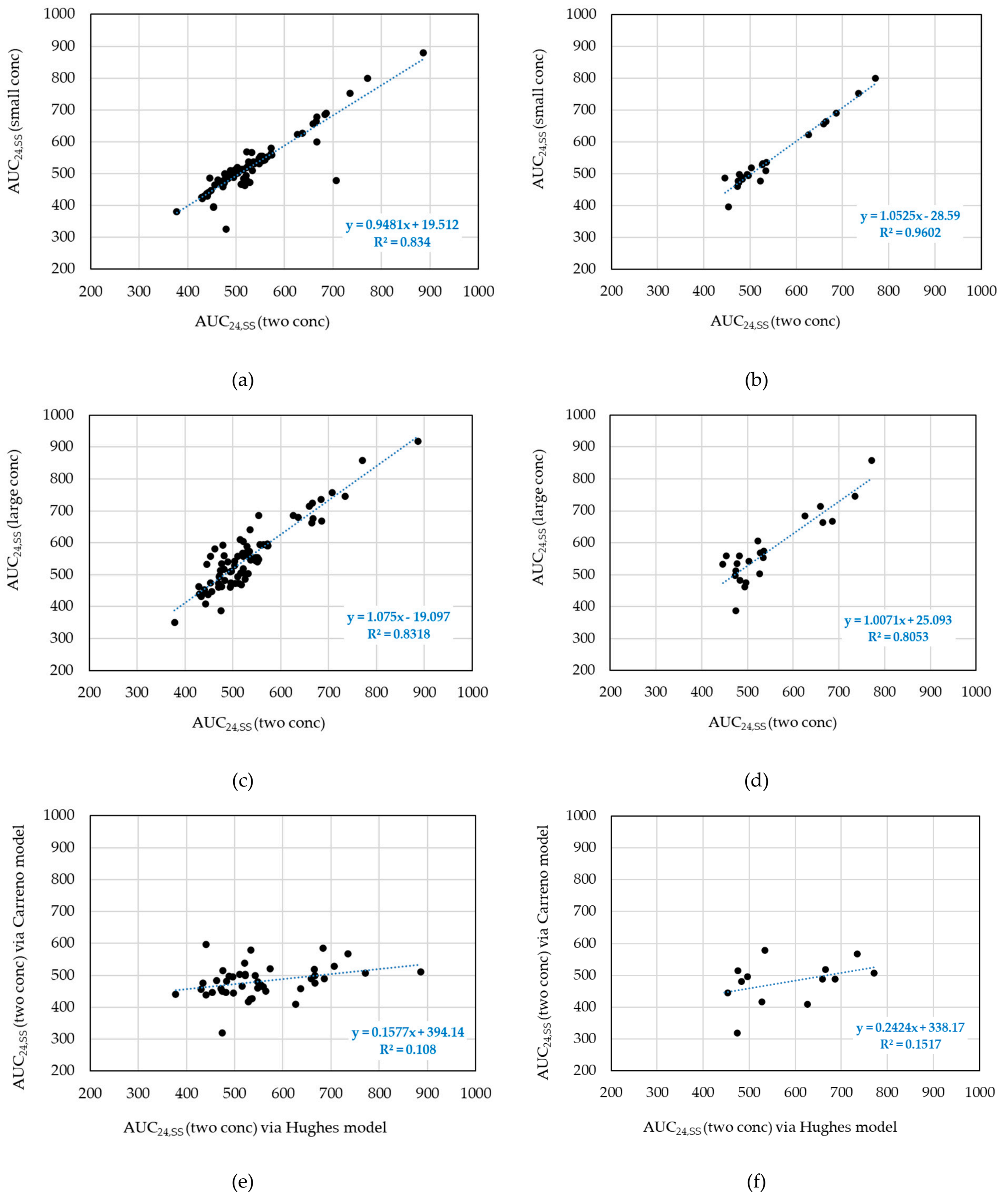

Figure S1: Comparison of $AUC_{24,SS}$ estimates obtained using different methods**.** Panels (a) and (b) compare small-concentration versus two-concentration $AUC_{24,SS}$ estimates in the full dataset and peak/trough subgroup, respectively. Panels (c) and (d) compare large-concentration versus two-concentration estimates in the same subgroups. Panels (e) and (f) compare two-concentration $AUC_{24,SS}$ estimates generated using the Carreno model versus the Hughes model in the same subgroup. Each point represents an individual dosing encounter, and the blue line shows the linear regression fit. The Hughes model was used for all AUC estimates in panels (a), (b), (c), and (d). Units of $AUC_{24,SS}$ are mg•h/L.

Small Concentration Fit Quality	Two Concentration Fit Quality: Good	Intermediate	Poor	Total
Good	62 (75.6%)	15 (18.3%)	3 (3.7%)	80 (97.6%)
Intermediate	1 (1.2%)	0 (0%)	0 (0%)	1 (1.2%)
Poor	0 (0%)	0 (0%)	1 (1.2%)	1 (1.2%)
Total	63 (76.8%)	15 (18.3%)	4 (4.9%)	

Figure S2: Distribution of the two-concentration and small-concentration fit quality assessments by the Bayesian software program across the categories of Good, Intermediate, and Poor. The distribution of fit quality assessments was significantly different by the McNemar-Bowker test (p = 0.0001). All Bayesian fits were completed with the Hughes model.

Large Concentration Fit Quality	Two Concentration Fit Quality: Good	Intermediate	Poor	Total
Good	62 (75.6%)	15 (18.3%)	1 (1.2%)	78 (95.1%)
Intermediate	1 (1.2%)	0 (0%)	3 (3.7%)	4 (4.9%)
Poor	0 (0%)	0 (0%)	0 (0%)	0 (0%)
Total	63 (76.8%)	15 (18.3%)	4 (4.9%)	

Figure S3: Distribution of the two-concentration and large-concentration fit quality assessments by the Bayesian software program across the categories of Good, Intermediate, and Poor. The distribution of fit quality assessments was significantly different by the McNemar-Bowker test (p = 0.005). All Bayesian fits were completed with the Hughes model.

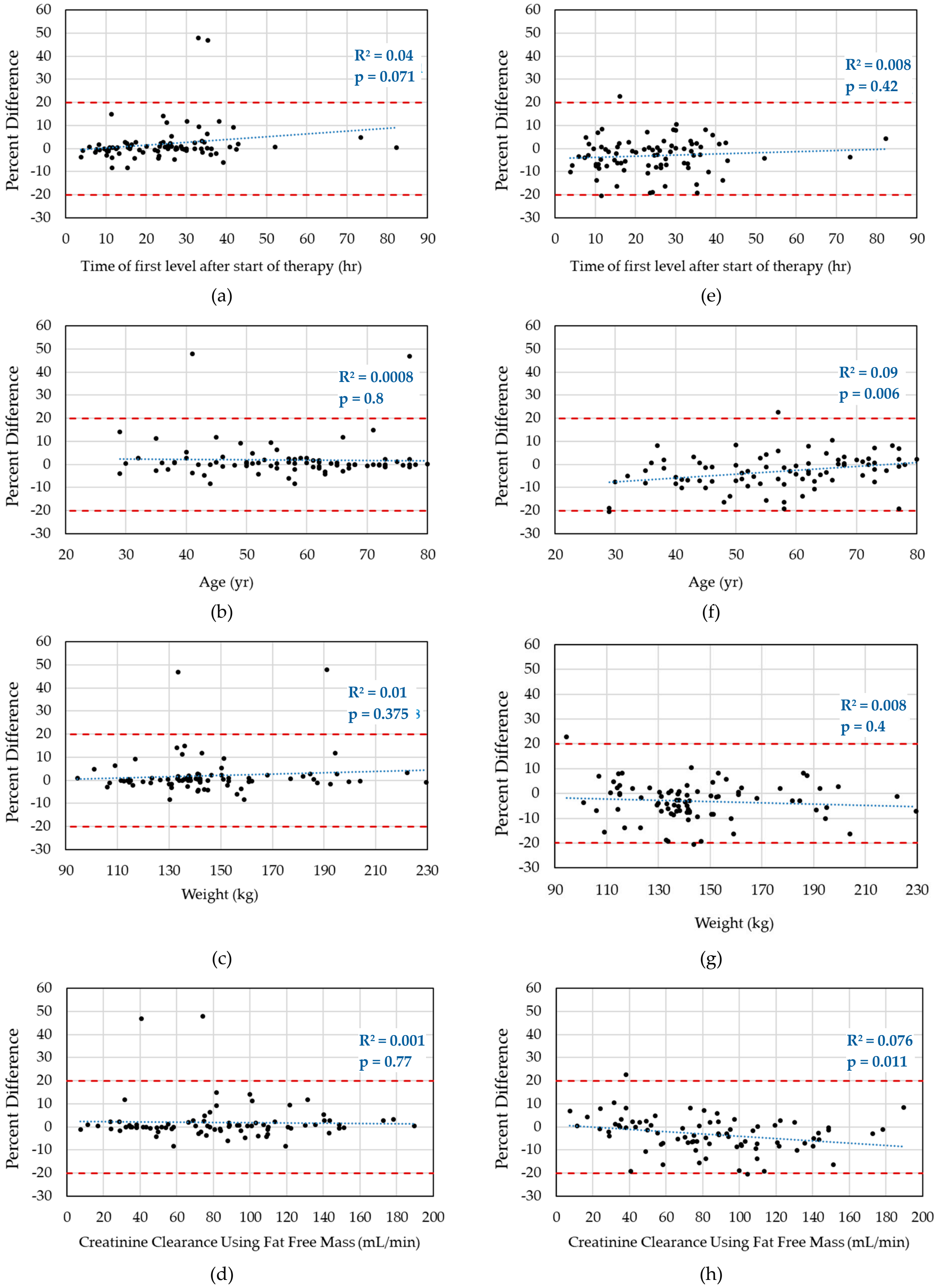

Figure S4: Percent difference between two-concentration and small-concentration $AUC_{24,SS}$ estimates (panels a, b, c, d) and between two-concentration and large-concentration $AUC_{24,SS}$ estimates (panels d, e, f, g) plotted as a function of the following variables: time from therapy initiation, age, weight, and creatinine clearance (N = 82 for all panels). Dashed red lines indicate the predefined ±20% range. The dashed blue line represents the linear regression fit; Pearson's R^2 is shown for each panel. The Hughes model was used for all AUC estimates.

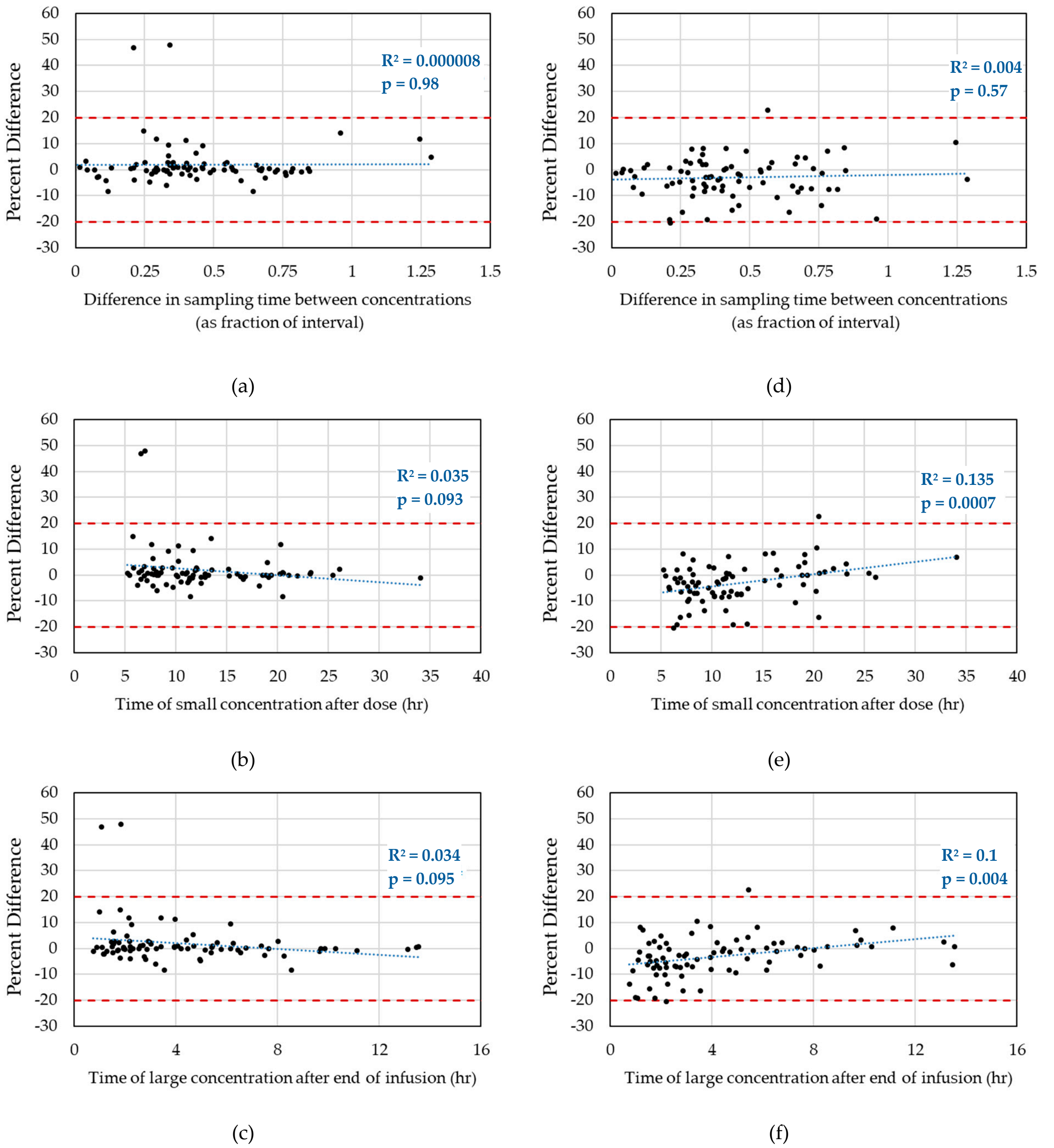

Figure S5: Percent difference between two-concentration and small-concentration $AUC_{24,SS}$ estimates (panels a, b, c) and between two-concentration and large-concentration $AUC_{24,SS}$ estimates (panels d, e, f) plotted as a function of the following variables: difference in sampling times as fraction of interval, time of small concentration after dose, and time of large concentration after dose (N = 82 for all panels). Dashed red lines indicate the predefined ±20% range. The dashed blue line represents the linear regression fit; Pearson's R^2 is shown for each panel. The Hughes model was used for all AUC estimates.

Table S1: Multivariable linear regression examining predictors of percent difference between two-concentration and small-concentration $AUC_{24,SS}$ estimates. The dependent variable was percent difference, with time of first measurement after initiation, time of trough after dose, and time of peak after dose included as independent variables.

Regression Statistics	
Multiple R	0.3
R Square	0.09
Adjusted R Square	0.05
Standard Error	8.14
Observations	82

ANOVA

	df	*SS*	*MS*	*F*	*Significance F*
Regression	3	496.31	165.44	2.5	0.066
Residual	78	5167.64	66.25		
Total	81	5663.95			

	Coefficients	*Standard Error*	*t Stat*	*P-value*	*Lower 95%*	*Upper 95%*
Intercept	2.96	2.73	1.09	0.28	-2.47	8.39
Time of first measurement after initiation (h)	0.13	0.07	1.93	0.06	-0.0042	0.26
Time of trough after dose (h)	-0.17	0.19	-0.92	0.36	-0.55	0.202
Time of peak after dose (h)	-0.33	0.35	-0.95	0.34	-1.02	0.36

FFM = fat free mass

Table S2: Multivariable linear regression examining predictors of percent difference between two-concentration and large-concentration $AUC_{24,SS}$ estimates. The dependent variable was percent difference, with age, creatinine clearance (CrCL, fat-free mass adjusted), time of trough after dose, and time of peak after dose included as independent variables.

Regression Statistics	
Multiple R	0.43
R Square	0.19
Adjusted R Square	0.15
Standard Error	6.89
Observations	82

ANOVA

	df	*SS*	*MS*	*F*	*Significance F*
Regression	4	848.94	212.24	4.47	0.00267
Residual	77	3658	47.51		
Total	81	4506.94			

	Coefficients	*Standard Error*	*t Stat*	*P-value*	*Lower 95%*	*Upper 95%*
Intercept	-14.93	6.02	-2.48	0.02	-26.92	-2.94
Age (yr)	0.11	0.07	1.63	0.11	-0.03	0.25
CrCL FFM (mL/min)	0	0.03	-0.01	0.99	-0.05	0.05
Time of trough after dose (h)	0.25	0.19	1.33	0.19	-0.13	0.63
Time of peak after dose (h)	0.4	0.29	1.37	0.17	-0.18	0.99

FFM = fat free mass
